# Structural mechanisms for α-conotoxin activity at the human α3β4 nicotinic acetylcholine receptor

**DOI:** 10.1038/srep45466

**Published:** 2017-03-31

**Authors:** Nikita Abraham, Michael Healy, Lotten Ragnarsson, Andreas Brust, Paul F. Alewood, Richard J. Lewis

**Affiliations:** 1 IMB Centre for Pain Research, Institute for Molecular Bioscience, The University of Queensland, St. Lucia, Queensland 4072, Australia

## Abstract

Nicotinic acetylcholine receptors (nAChR) are therapeutic targets for a range of human diseases. α-Conotoxins are naturally occurring peptide antagonists of nAChRs that have been used as pharmacological probes and investigated as drug leads for nAChR related disorders. However, α-conotoxin interactions have been mostly characterised at the α7 and α3β2 nAChRs, with interactions at other subtypes poorly understood. This study provides novel structural insights into the molecular basis for α-conotoxin activity at α3β4 nAChR, a therapeutic target where subtype specific antagonists have potential to treat nicotine addiction and lung cancer. A co-crystal structure of α-conotoxin LsIA with *Lymnaea stagnalis* acetylcholine binding protein guided the design and functional characterisations of LsIA analogues that identified the minimum pharmacophore regulating α3β4 antagonism. Interactions of the LsIA R10F with β4 K57 and the conserved –NN– α-conotoxin motif with β4 I77 and I109 conferred α3β4 activity to the otherwise inactive LsIA. Using these structural insights, we designed LsIA analogues with α3β4 activity. This new understanding of the structural basis of protein-protein interactions between α-conotoxins and α3β4 may help rationally guide the development of α3β4 selective antagonists with therapeutic potential.

Neuronal nicotinic acetylcholine receptors (nAChRs) are ligand gated ion channels involved in the modulation of neurotransmission in the central and peripheral nervous system^1^,^2^,^3^,^4^. The nAChR subtypes can be homopentamers such as the α7 and α9 or heteropentamers composed of a combination of α (α2–α10) and β (β2–β4) subunits such as the α3β2 and α3β4 subtypes^1^,^2^. nAChRs are associated with Alzheimer’s, Parkinson’s and schizophrenia^3^,^5^,^6^,^7^ and the therapeutic potential of nAChRs rests on the ability to develop subtype selective modulators that can define the relative role of each of the different subtypes in normal and disease processes^8^. Many of the available plant and animal toxins have naturally engineered specificity for the α1β1γδ/ε (muscle), α7 and α3β2 (neuronal) subtypes^9^,^10^,^11^. This provided opportunities for several detailed investigations into the ligand recognition and selectivity mechanisms at these subtypes, providing the framework required for the rational development of therapeutics^12^,^13^,^14^,^15^,^16^,^17^,^18^. In comparison, such detailed structural and functional characterisations of the α3β4 activity are currently lacking. Primarily due to the small number of α3β4 specific peptides (Table 1). α3β4 is the predominant nAChR subtype in the autonomic nervous system, contributing to the “reward” sensation associated with nicotine addiction and drug abuse as well as the development and progression of lung cancer^19^,^20^,^21^. Thus antagonists of the α3β4 nAChR may have anti-addictive and anti-cancer potential.

α-Conotoxins are a large family of disulfide rich peptide antagonists of the nAChRs isolated from the venom of marine cone snails^11^,^22^. Over fifty α-conotoxins have been isolated and characterised to date^11^, including a small number with α3β4 activity (Table 1). To broaden our understanding of nAChR pharmacology, we used α-conotoxin LsIA to identify the minimum structural requirements for α-conotoxin activity at human α3β4 nAChR. Native LsIA is an equipotent antagonist of the human α7 and rat α3β2 but inactive at α3β4 nAChRs^23^ despite relatively high sequence identity to α-conotoxins with activity at the α3β4 nAChRs^23^,^24^,^25^,^26^,^27^,^28^,^29^,^30^,^31^,^32^ (Table 1). Using a co-crystal structure of LsIA and *Lymnaea stagnalis* acetylcholine binding protein (AChBP) to guide mutational studies, we identified that LsIA arginine at position 10 (R10) and asparagine at position 12 (N12) determined LsIA inactivity at α3β4. Systematic modifications of these positions allowed us to rationally design LsIA analogues with enhanced α3β4 subtype activity. Our data reveals a β4 triad comprising K57, I77 and I109 that represents a minimum pharmacophore for α-conotoxins inhibition of α3β4.

## Results

### Crystal structure of LsIA in complex with Ls-AChBP

**Figure 1:**
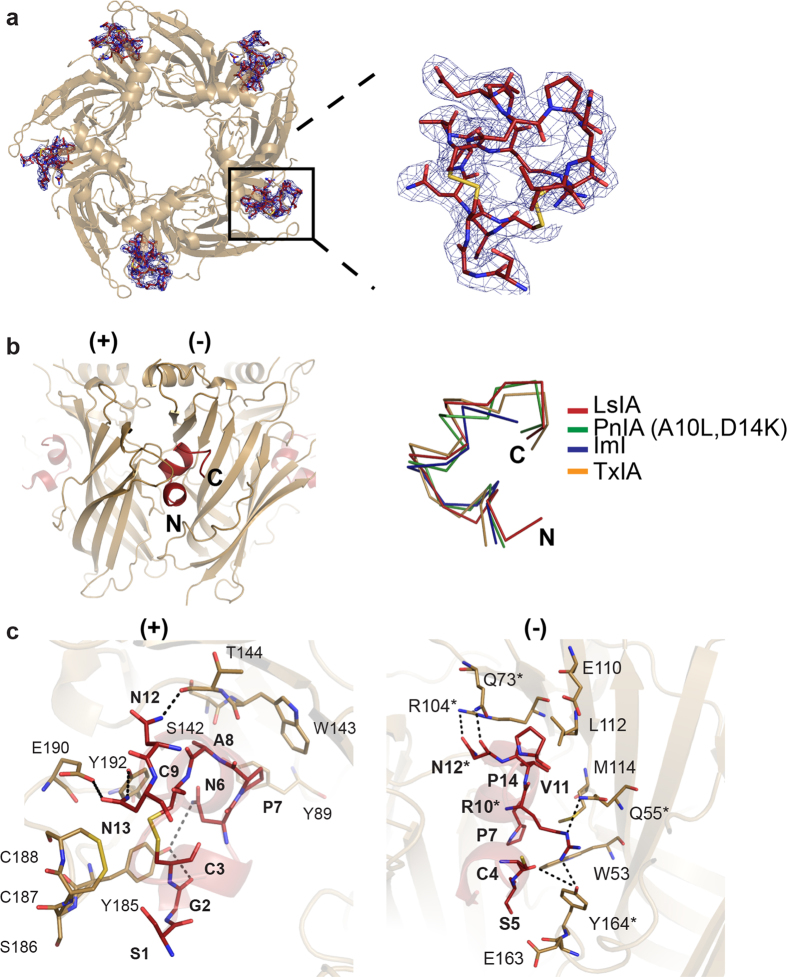
LsIA/ Ls-AChBP co-crystal structure. (**a**) LsIA-NH2 was co-crystallised with Ls-AChBP. Clear electron density for the ligand was seen in all five binding pockets.(2Fo-Fc) map for the ligand countoured to 1.0 σ is shown. (**b**) LsIA binds to the orthosteric binding pocket with the α-helical backbone buried deep within the pocket, the N-terminus oriented to the bottom and C-terminus to the top of the pocket. Within the binding pocket LsIA adopts the typical α-conotoxin binding orientation, as can be seen from the superimposition of LsIA backbone with that of previously co-crystallised α-conotoxins PnIA(A10L,D14K), ImI and TxIA. (**c**) The receptor ligand interactions are characterised by several hydrogen bonds and some hydrophobic interactions (dotted lines indicate hydrogen bonds). Interactions LsIA R10 and N12 (*) were investigated in this study. These interactions were found to be important for LsIA activity at the α3β4 subtype.

The structure of the LsIA and Ls-AChBP complex was determined at 2.8 Å and solved using molecular replacement (Fig. 1a and b). The diffraction data and electron density maps were well defined except for certain residues on flexible loops (mostly AChBP loop F) that were excluded from the final model. The asymmetric unit contains one pentamer with LsIA bound to all five binding pockets. The final structure was refined to an *R*_*free*_ value of 0.24 (Supplementary Table S1)

LsIA retains the typical α-conotoxin binding orientation with solvent exposed N- and C-termini oriented to the bottom and the top of the pocket respectively and the α-helical backbone buried into the binding pocket. (Fig. 1b). The C loop of Ls-AChBP is displaced outward by a distance of 10.54 ± 0.20 Å as measured between the Cys 187 C_α_ atom in Ls-AChBP/LsIA and the HEPES-bound Ls-AChBP structure, similar to other α-conotoxin complexes^13^,^14^,^17^. Pair-wise interactions of LsIA with AChBP consisted of a combination of conserved α-conotoxin interactions and several interactions unique to the LsIA/Ls-AChBP complex (Fig. 1c, Supplementary Table S2). LsIA differs only by four residues from the majority of α-conotoxins active at the α3β4. The variable residues include LsIA S1, N6, R10 and N15 (Table 1). Pair-wise interactions of these residue were closely inspected to determine their contributions to the LsIA pharmacological profile at the nAChRs. LsIA S1 was found to be highly flexible and solvent exposed. LsIA N6 interacts with residues on the plus face of the binding pocket that are highly conserved across the different nAChR subtypes. LsIA N15 forms part of the solvent exposed C-terminus and the co-crystal structure does not reveal any significant interactions of this residue. Interestingly, LsIA R10 forms unique hydrogen bonds with AChBP Q55 (3.2 ± 0.11 Å) and Y164 (3.2 ± 0.26 Å) on the minus face of the ligand binding pocket (Fig. 1c). In addition to the interactions of the variable residues, unique interactions of LsIA N12 with Q73 (2.9 ± 0.05 Å), R104 (3.2 ± 0.07 Å) on the variable minus face^(16,37)^ of the ligand binding pocket were also observed (Fig. 1c). LsIA N12 is reasonably well conserved between LsIA and α-conotoxins active at the α3β4 (Table 1), and therefore is unlikely to be responsible for LsIA inactivity at α3β4 nAChR. Regardless, hydrogen bonds of LsIA N12 were consistently observed in all five binding pockets, suggesting an important role of this residue in LsIA activity.

The unique interactions of LsIA R10 and N12 with the complementary face of the pocket were expected to have important influences on α3β4 activity and were investigated further.

### Unique interactions of LsIA R10 and N12 can influence activity at α3β4 nAChR

**Figure 2:**
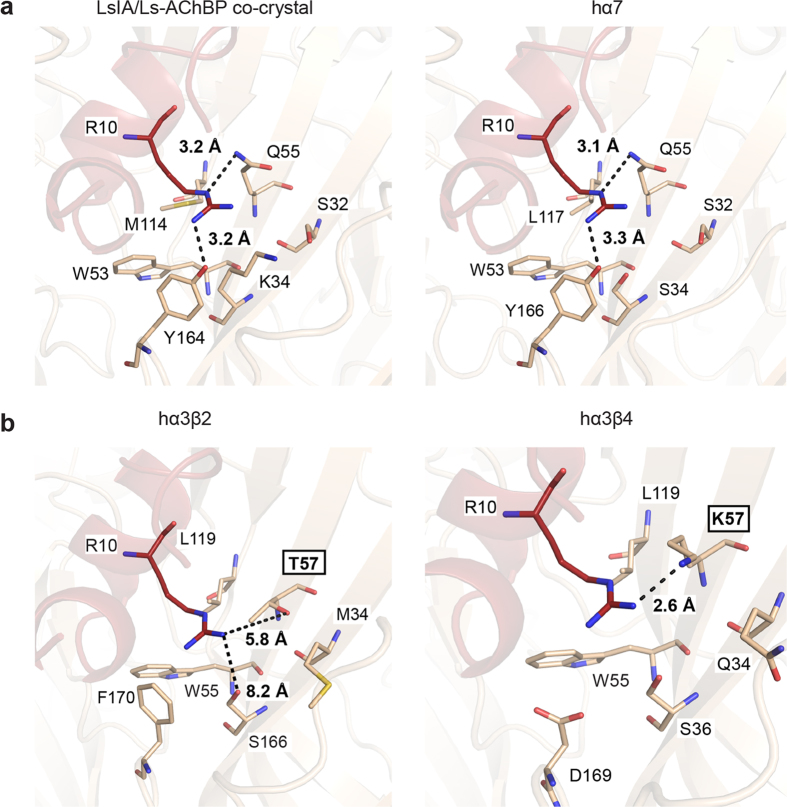
LsIA R10 interactions at the human α7, α3β2 and α3β4 nAChR subtypes. (**a**) The homology model of the α7 receptor was generated based on the Ls-AChBP/LsIA co-crystal structure. The residues constituting the surface interacting with LsIA R10 are similar in both the α7 and the Ls-AChBP. Therefore, it is likely that the LsIA R10 engages in interactions similar to those seen in the crystal structure. (**b**) Residues on the human α3β2 and α3β4 subtype forming the surface that interacts with the LsIA R10 are shown. The interacting surface on α3β2 consists of hydrophobic residues with the exception of T57 and S166, which are outside of hydrogen bonding distance. On the α3β4 interacting surface residue K57 lies in close proximity (2.6 Å) to LsIA R10. This is thought to contribute to the inactivity of LsIA at this subtype.

**Figure 3:**
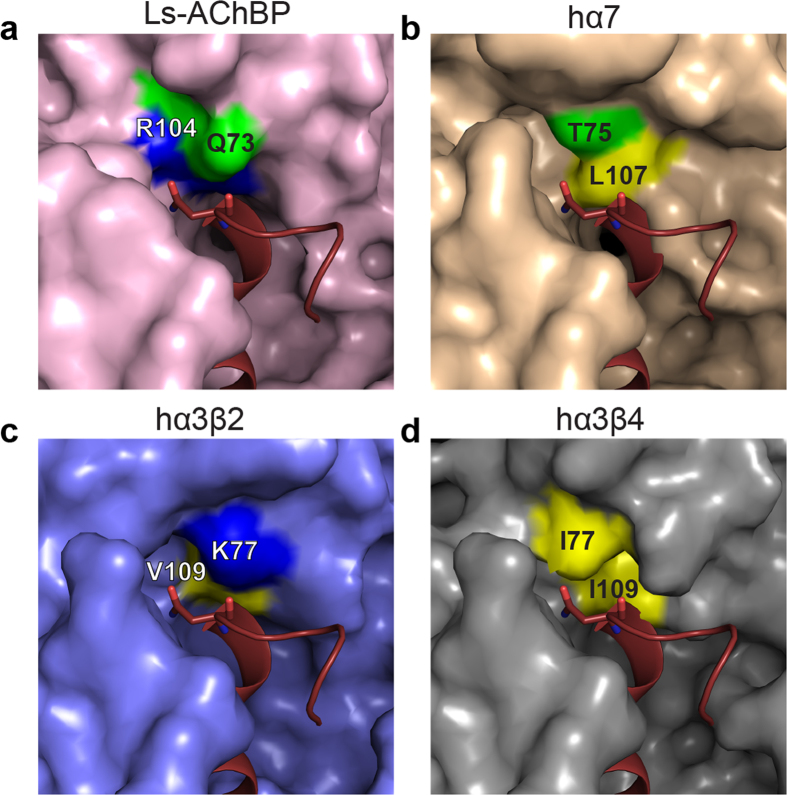
LsIA N12 interactions at Ls-AChBP and the human α7, α3β2 and α3β4. Residues constituting the interacting surface for LsIA N12 on Ls-AChBP as seen in the structure and the corresponding residues in α7, α3β2 and α3β4 are shown. The more extensive hydrophobic patch on α3β4 contributes to the enhanced affinity of the N12L analogue at this subtype.

To determine the contribution of R10 and N12 interactions to LsIA activity, homology models of α7, α3β2 and α3β4 nAChRs bound to LsIA were generated using the LsIA/Ls-AChBP co-crystal structure. A comparison of the three homology models were used to infer the implications of the R10 and N12 interactions on α3β4 activity (Figs 2 and 3).

#### LsIA R10 interactions

The surface interacting with LsIA R10 in the co-crystal structure consists of non-polar W53, M114, charged K34, D160, polar residues S32, Q55, and Y164 where the LsIA R10 engages in hydrogen bonds with Q55 (3.2 ± 0.11 Å) and Y164 (3.2 ± 0.26 Å). Likewise, in the α7 nAChR homology model, the LsIA R10 interacting surface is comparable to that seen in the co-crystal structure where it comprises non-polar W53, L117 and polar S32, S34, Q55, and Y166 (Fig. 2a). Therefore, interactions similar to those seen in the co-crystal structure were expected between LsIA and the α7 nAChR, including hydrogen bonds of LsIA R10 with Q55 (3.1 ± 0.12 Å) and Y166 (3.3 ± 0.32 Å) on the α7. In contrast, the α3β2 model has a corresponding interacting surface that is relatively more hydrophobic, constituting residues M34, W55, L119, F170 and polar T57, S166 (Fig. 2b). Therefore, this surface on α3β2 is not favourable to engage LsIA R10 in polar interactions and does not show any other significant interactions. Lastly, the interacting surface on the α3β4 model consists of polar residues Q34, S36, W55, charged K57, D169 and non-polar L119. The α3β4 K57 is in close proximity (2.6 Å) to LsIA R10 and can potentially introduce an electrostatic clash (Fig. 2b).

#### LsIA N12 interactions

In the LsIA/Ls-AChBP co-crystal structure, LsIA N12 interacts with a polar surface constituting Q73, S75 and R104 and engages two of these residues, Q73 (2.9 ± 0.05 Å) and R104 (3.2 ± 0.07 Å) in hydrogen bonds (Fig. 3a). The corresponding surfaces on the α7, α3β2 and α3β4 models do not present favourable interactions with LsIA N12. In the α7 homology model, the surface comprises non-polar L107 and polar T75 that is outside hydrogen bonding distance (5.6 Å) (Fig. 3b). Likewise, in the α3β2 corresponding residues include non-polar V109 and positively charged K77 outside of hydrogen bonding distance (4.8 Å) (Fig. 3c). Most notably, in the α3β4 model, the corresponding surface is strongly hydrophobic, and therefore is unlikely to offer favourable interactions for the polar LsIA N12, as seen in the Ls-AChBP co-crystal structure (Fig. 3d).

The LsIA/Ls-AChBP co-crystal structure along with the homology models suggested that both LsIA R10 and N12 present unfavourable interactions at the α3β4 subtype and can potentially be responsible for the inactivity of LsIA at the α3β4 nAChR.

### LsIA analogues

To functionally validate observations from the co-crystal structure and nAChR homology models, LsIA analogues with modifications at the R10 and N12 positions were synthesised. The arginine residue (R10) in LsIA was substituted with residues presenting different functional groups with the aim of removing the potential electrostatic clash between LsIA R10 and α3β4 K57. These included a hydrophobic methionine (R10M-LsIA), charged aspartic acid (R10D-LsIA) and aromatic phenylalanine (R10F-LsIA). Additionally, R10D-LsIA and R10F-LsIA were expected to introduce favourable interactions with α3β4 K57 through a salt bridge and cation-π, respectively (Fig. 2b). Similarly, LsIA N12 was substituted with glutamine (N12Q-LsIA), aspartic acid (N12D-LsIA) and leucine (N12L-LsIA) to determine the influence of side chain length and different functional groups on LsIA activity. N12L-LsIA was specifically generated to introduce favourable interactions with the hydrophobic patch on the α3β4 as seen in the homology model (Fig. 3c).

An additional analogue was synthesised, where LsIA asparagine at position 6 was substituted to a histidine (N6H-LsIA). The LsIA/Ls-AChBP co-crysal structure does not reveal any interactions of LsIA-N6 that can be significant for activity at the α3β4 nAChR. However, a histidine at position 6 (LsIA numbering) is seen in a majority of α-conotoxins active at α3β4. Therefore N6H-LsIA analogue was generated to investigate the role of this histidine residue in ligand recognition at the α3β4 receptor. Circular dichroism (CD) was used to confirm the structural integrity of the chemically synthesised LsIA and the analogues (Supplementary Fig. S1). The CD spectroscopy profile for LsIA and analogues were consistent with that expected for an α-helical structure with the exception of R10D-LsIA. The overall circular dichroism profile for R10D-LsIA remained consistent with that expected for an α-helical peptide. However, the peak at 222 nm was shallower and the peak at 204 nm shifted to 200 nm as compared to LsIA (Supplementary Fig. S1), suggesting that R10D-LsIA conformation might differ from the conformation of native LsIA, possibly due to global structural changes in the peptide induced by this modification.

### Characterisation of the binding profile of LsIA analogs to AChBPs

**Figure 4:**
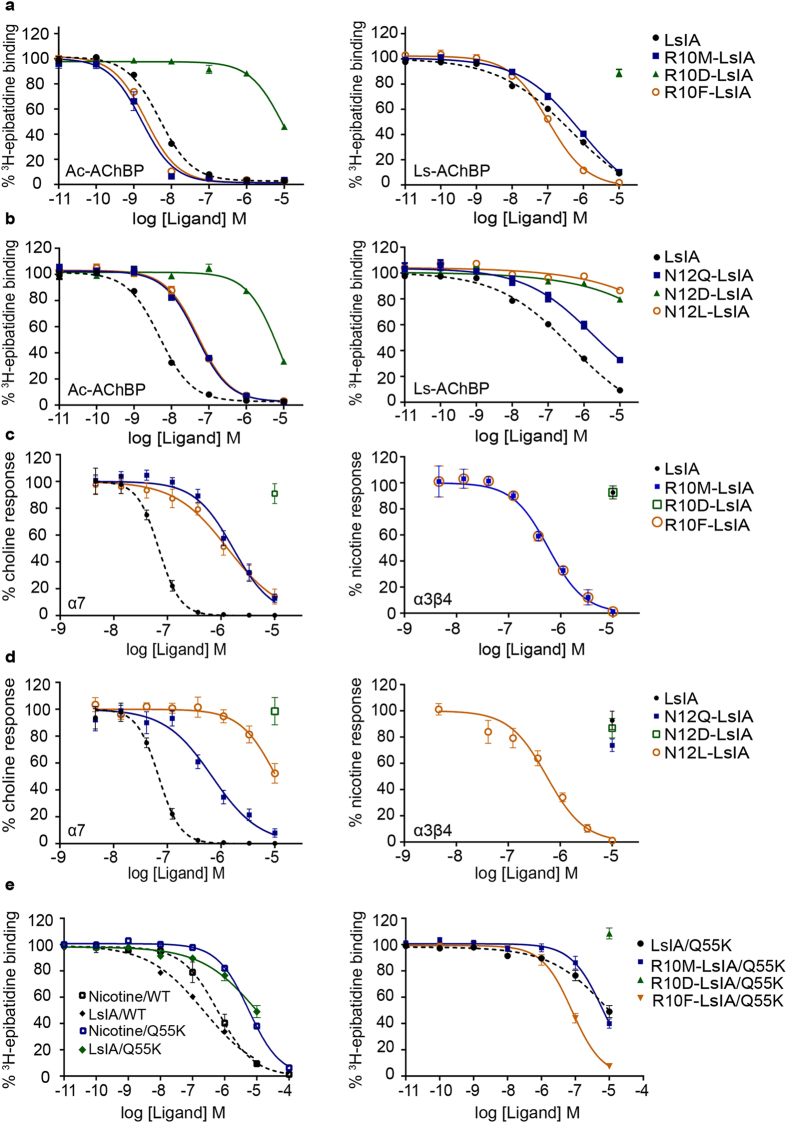
Functional characterisation of LsIA analogues at AChBPs, α7 and α3β4 nAChRs and Q55K mutant AChBP. (**a,b**) Displacement of ^3^H-epibatidine from Ac and Ls-AChBP by R10 and N12 analogues of LsIA. (**c,d**) Concentration response curves for LsIA analogues at the α7 and α3β4 nAChRs. (**e**) Displacement of ^3^H-epibatidine from Q55K mutant Ls-AChBP by LsIA and LsIA-R10 analogues. Data represent the mean ± S.E.M of triplicate data from three independent experiments.

The binding of the LsIA analogues were tested on Ls- and Ac-AChBPs to validate the functional implications of unique interactions seen in the co-crystal structure. Since Ls and Ac-AChBPs have characteristic binding profiles, LsIA analogues were characterised at both the AChBP species to assess the contribution of these mutations to peptide selectivity profile. LsIA analogues of positions 10 and 12 differentially affected affinity at the two AChBP species (Fig. 4a and b). R10M-LsIA and R10F-LsIA had slightly improved affinities at Ac-AChBP (2.8 and 2.4-fold respectively), whereas at Ls-AChBP the R10M-LsIA had about 2.4-fold lower affinity and R10F-LsIA had 2.0-fold higher affinity than LsIA. The R10D-LsIA had >1000-fold lower affinity than LsIA at Ac-AChBP and was inactive at Ls-AChBP (Fig. 4a and Table 2). The LsIA R10 interacting surface on Ls-AChBP more closely resembles the α7 surface than that on Ac-AChBP (Supplementary Fig. S7). Therefore, as expected disruption of the dominant polar interactions with S32, Q55 and Y164 as seen in the co-crystal structure results in a loss of affinity for R10M-LsIA at Ls-AChBP. While the R10F-LsIA would also disrupt such polar interactions, the affinity could perhaps be sustained by aromatic interactions between R10F and W53 and M114.

In contrast, the N12 substitutions resulted in a loss of affinity compared to LsIA at both AChBP species (Fig. 4b). N12Q-LsIA had a 10-fold reduced affinity, while the N12D substitution caused >2,500 fold loss in affinity at both AChBPs. Interestingly, N12L-LsIA caused a complete loss of activity at Ls-AChBP and a ~ 10-fold reduction of affinity at Ac-AChBP (Table 2). Neither AChBPs mimic the hydrophobic I77 and I109 on α3β4, which could explain the significant loss in affinity for the N12L analogue Therefore, reinforcing the conclusion from the homology modelling, that this position is a key α3β4 activity determinant. Thus binding studies confirmed that modifications of LsIA at R10 and N12 have the capacity to influence peptide activity.

### Characterisation of LsIA analogues at α7, α3β4 nAChRs expressed in SH-SY5Y cells

Functional profiles of LsIA R10 and N12 analogues were characterised on SH-SY5Y cells that endogenously express the human α7 and α3β4 nAChRs^33^,^34^,^35^,^36^. LsIA had an IC_50_ of 70 ± 7 nM at human α7 but was inactive at human α3β4 up to 10 μM (Fig. 4c and d and Table 2), consistent with its selectivity at the highly homologous rat nAChR subtypes^23^ (Supplementary Figs S2, S3, S4 and S5). R10M-LsIA and R10F-LsIA substitutions were 35- and 6-fold less potent than LsIA, respectively, while the R10D-LsIA was inactive at the α7 receptor, consistent with homology models that suggest a disruption of polar interactions between R10 and the α7 receptor (Figs 2a and 4c). In contrast, removing the potential clash observed in the homology model between LsIA R10 and α3β4 K57, by introducing smaller, uncharged residues in R10M-LsIA and R10F-LsIA conferred α3β4 activity to the peptide (Figs 2b and 4c). The R10M-LsIA and R10F-LsIA analogues were equipotent at the α3β4 (IC_50_ of 0.44 ± 0.13 μM). The R10F-LsIA analogue was equipotent at both the α7 and α3β4 nAChRs, whereas R10M-LsIA was 5-fold more potent at the α3β4 receptor. The R10D-LsIA was inactive at both subtypes (upto 10 μM), possibly due to an altered peptide conformation as suggested by CD (Supplementary Fig. S1) and was considered unsuitable for inferring the absence of a predicted pairwise interaction between the D10 on R10D-LsIA and K57 on the α3β4 nAChR.

Substitutions of LsIA N12 also led to a reduction of peptide potency at the α7 (Fig. 4d and Table 2). The N12Q-LsIA and N12L-LsIA were 15-fold and 400-fold less potent, whereas the N12D-LsIA substitution had no significant activity up to 10 μM. At the α3β4, the N12L-LsIA was designed to specifically interact with the hydrophobic patch observed in the α3β4 homology model (Fig. 3d). Consistent with this, N12L-LsIA was the only active analogue, confirming that the hydrophobic patch on α3β4 does indeed contribute to ligand recognition at the α3β4. In fact, the N12L-LsIA was 40-fold more selective for the α3β4 over the α7 (Fig. 4d and Table 2). The N6H-LsIA analogue did not affect potency at α7 and was inactive at the α3β4 nAChR, suggesting that the conserved histidine found in other α-conotoxins is not a primary determinant of α3β4 activity (Supplementary Fig. S6 and Table 2).

### Characterisation of LsIA and LsIA R10 analogues at Q55K Ls-AChBP mutant

The modelling and functional characterisations established the unfavourable interaction between LsIA R10 and K57 on the β4 subunit as a key factor for inactivity of LsIA at the α3β4 nAChR (Figs 2b and 4c). To further validate the role of K57 and to determine the degree of influence of an electrostatic repulsion on peptide activity, we introduced a lysine residue at the equivalent position in Ls-AChBP (Ls-AChBP Q55 is equivalent of the β4 K57). As predicted, LsIA binding affinity was reduced by ~100-fold (Table 2 and Fig. 4e) at the Q55K mutant confirming that an unfavourable interaction such as an electrostatic clash can significantly affect peptide activity. Interestingly, the R10F-LsIA analog restored affinity at Q55K Ls-AChBP with an IC_50_ value of 0.8 ± 0.26 μM, equivalent to the affinity at wt Ls-AChBP (0.21 ± 0.04 μM) (Table 2). Depending on the orientation of the side chains, favourable interactions for example a cation-π interaction between the aromatic ring of the phenylalanine in R10F-LsIA and the cationic core of K57 on β4 can explain the enhanced affinity observed. In contrast, the R10D-LsIA was inactive at the Q55K Ls-AChBP and R10M-LsIA had 15-fold reduced affinity at Q55K Ls-AChBP than at wt AChBP (Table 2 and Fig. 4e).

Finally, we attempted to mutate residues Ls-AChBP R104 and Q73 to mimic the hydrophobic patch on the α3β4 formed by I77 and I109 to confirm the contribution of LsIA N12 to LsIA inactivity at the α3β4. However, the mutated protein failed to express and purify stably, suggesting these residues may play a structural role.

### [R10F][N12L]-LsIA double mutant shifts selectivity from α7 towards α3β4 nAChR

**Figure 5:**
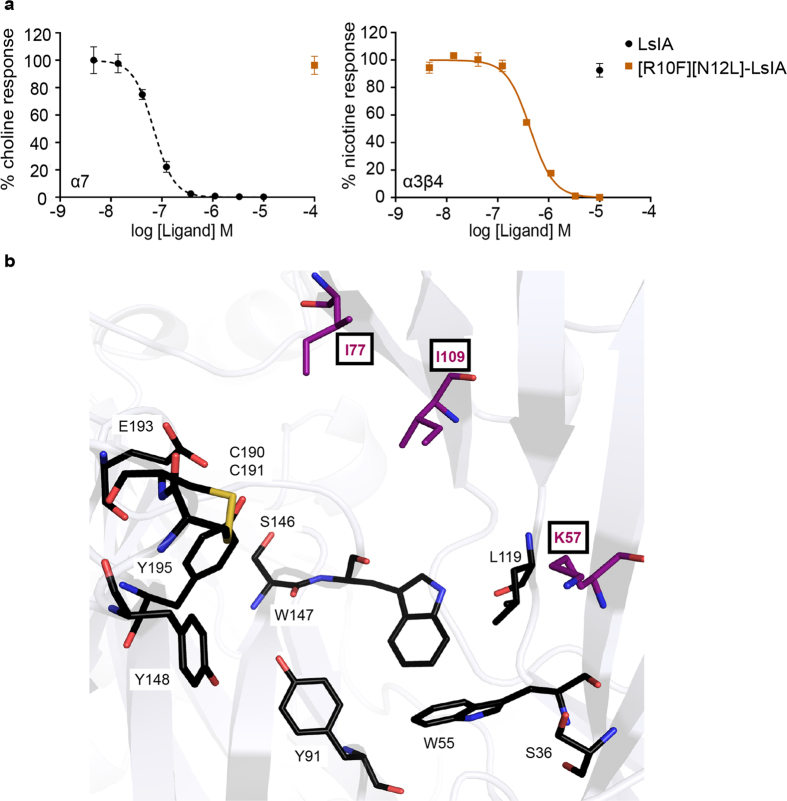
α3β4 pharmacophore. (**a**) α3β4 activity was successfully introduced into LsIA through systematic modification of interactions at position 10 and 12. The [R10F][N12L]-LsIA provided a > 250-fold selectivity for α3β4 over α7 nAChR. Data represent the mean ± S.E.M of triplicate data from three independent experiments. (**b**) The highly conserved aromatic cage involved in ligand recognition at the nAChRs is shown in black. Using α-conotoxin LsIA we have identified residues (boxed) that lie outside this conserved aromatic cage and contribute to ligand recognition at the α3β4.

The functional profiles of the single mutants demonstrated the contribution of both the R10F and N12L interactions to LsIA activity at the α3β4 nAChR. Consistent with this, the [R10F][N12L]-LsIA double mutant selectively inhibited the α3β4 nAChR with an IC_50_ of 0.41 ± 0.02 μM, whereas no significant inhibition was observed at the α7 up to 100 μM (Fig. 5 and Table 2).

## Discussion

Antagonists of the α3β4 are required to better characterise the role of this subtype in physiology and disease and have the potential as therapeutic leads for nicotine addiction and lung cancer^20^,^21^. A small number of α-conotoxins with α3β4 activity have been discovered^19^,^20^,^21^,^22^,^23^,^24^,^25^, presenting promising leads for the development of α3β4 selective inhibitors. However, key receptor-ligand interactions required for rational development of inhibitors have not been extensively characterised. In this study we attempted to fill this gap by determining the structural determinants of α-conotoxin activity at α3β4.

α-Conotoxin LsIA has high sequence identity to α3β4 active α-conotoxins but does not inhibit this subtype, making it useful to determine the minimum pharmacophore required for α3β4 antagonism. The 2.8 Å co-crystal structure of Ls-AChBP with bound LsIA revealed unique interactions of LsIA R10 and N12 that could potentially contribute to this pharmacology. LsIA R10 participates in a unique hydrogen bond network with residues on the minus face of the ligand binding pocket, which is largely responsible for α-conotoxin activity and selectivity at nAChRs^16^,^37^. The α3β4 homology model built from the Ls-AChBP crystal structure revealed that the orientation of the positively charged LsIA R10 likely introduces unfavourable interactions such as an electrostatic clash with the positively charged K57 on the β4 subunit to further reduce affinity at this subtype. In support, R10M-LsIA and R10F-LsIA analogues with uncharged side chains at this position conferred α3β4 activity to LsIA. The contribution of α3β4 K57 to this predicted clash was confirmed by introducing this residue in the equivalent position in Ls-AChBP (Q55K Ls-AChBP), which resulted in a 100-fold drop in LsIA potency. Therefore, such an interaction between LsIA R10 and K57 of the α3β4, as seen in the homology models, can indeed be a key factor contributing to the inactivity of LsIA at α3β4. Characterisation of the LsIA R10 analogues at Q55K Ls-AChBP revealed enhanced affinity for the R10F-LsIA analogue, indicating that replacing LsIA R10 with an aromatic residue likely establishes favourable interactions, possibly a cation-π interaction with the α3β4 K57 depending on the orientation of the side chains. Interestingly, previous mutagenesis studies on α-conotoxins MII and BuIA have suggested that β4 K57 contributes to α-conotoxin selectivity differences between α3β2 and α3β4^38^. A similar trend is observed with α3β4 specific RegIIA^17^, BuIA^19^ and TxID^23^, which have uncharged shorter side chains, that unlike LsIA R10 would not present unfavourable interactions with K57. Therefore, further reinforcing the role of R10 in determining LsIA inactivity at α3β4.Thus, interactions with α3β4 K57 revealed through these LsIA analogues are reflective of a general mechanism modulating α-conotoxin activity at this subtype.

The next novel interactions investigated from the co-crystal structure were a set of hydrogen bonds observed between LsIA N12 and Q73 (2.9 ± 0.05 Å) and R104 (3.2 ± 0.07 Å) on Ls-AChBP. LsIA N12 is the first residue of a –NN– motif that is conserved in many α-conotoxins^24^,^26^,^27^,^28^,^29^,^30^,^31^,^32^ and interactions of this residue with the conserved plus face of the ligand pocket have been identified in previous co-crystal structures^13^,^14^,^17^. However, interactions of this residue with the variable minus face of the ligand binding pocket were observed for the first time in our LsIA/Ls-AChBP co-crystal structure. While LsIA N12 was considered unlikely to contribute to the inactivity of LsIA at α3β4, since it is conserved in majority of the α3β4 specific α-conotoxins, unique interactions with the minus face warranted further investigation. Homology modelling demonstrated that the α3β4 surface interacting with LsIA N12 comprised the hydrophobic residues I77 and I109. As expected, inducing favourable hydrophobic interactions with the N12L-LsIA analogue enhanced α3β4 activity but also reduced α7 activity, to yield a 40-fold selectivity window. The significance of these hydrophobic interactions are supported by recent mutagenesis studies, where substitution of the –NN– motif with alanine (–AA–) significantly reduced α-conotoxin RegIIA potency at α7 and α3β2 nAChR but not α3β4, thereby enhancing selectivity for α3β4^25^. Our structure-activity studies show that it is essentially the first asparagine in the conserved –NN– motif that influences activity. Systematic modification of this asparagine are expected to be sufficient to improve the selectivity and maintain the potency of α-conotoxins at the α3β4 nAChR.

In order to define the minimum requirements of antagonist recognition at the α3β4 subtype, we investigated the contribution of asparagine at position 6 in LsIA (N6). In the LsIA/Ls-AChBP co-crystal structure, LsIA N6 interacts with AChBP Y185, which is conserved across the nAChR subtypes and therefore not expected to influence subtype selectivity. However, LsIA N6 is replaced by histidine in most α-conotoxins active at the α3β4 nAChR, suggesting histidine at this position might also contribute to α3β4 activity. However, the N6H-LsIA failed to confer α3β4 activity to LsIA, showing that histidine at this position is not sufficient for α3β4 activity.

The interactions of LsIA R10F with β4 K57 and N12L with the hydrophobic patch comprising I77 and 109 appear as the primary determinants of LsIA activity at the α3β4 subtype. The [R10F][N12L]-LsIA double mutant supports this conclusion, since α3β4 nAChR affinity is maintained at the expense of α7 affinity to provide >250-fold selectivity for α3β4 over α7 nAChR. A comparison of the α3β4, α7, α3β2 and α4β2 residues equivalent to the α3β4 triad reveal significant sequence variations (Supplementary Fig. S9). Therefore, in addition to being critical for α3β4 activity, the K57, I77 and I109 triad could potentially have secondary effects on subtype selectivity. Interestingly, despite the dramatic selectivity for α3β4 over α7, there is no improvement in affinity for the LsIA double mutant versus the single mutant. A co-crystal structure of [R10F][N12L]-LsIA with AChBP could determine whether this interesting pharmacology arises from a difference in binding modes between the double and single mutants. The identified β4 K57, I77 and I109 triad also potentially underpins the activity of α-conotoxin AuIB, a commonly used α3β4 probe and TP-2212-59, the most potent α3β4 antagonist identified from a chemical combinatorial library^20^,^31^. Both AuIB and TP-2212-59 possess an aromatic phenylalanine at position 10 (LsIA numbering) that could favourably interact with α3β4 K57. Additionally, TP-2212-59 contains a hydrophobic norvaline at position 12 (LsIA numbering) that can favourably interact with α3β4 I77 and I109. Building on these observations, we predict that the selectivity of α-conotoxin AuIB could be improved by the substitution of the threonine at position 12 with a hydrophobic residue.

In conclusion, our structure-function studies show that α-conotoxin interactions with the β4 K57, I77 and I109 triad forms the minimum pharmacophore required for α3β4 inhibition. Interactions of α-conotoxin residues at position 10 (LsIA numbering) with α3β4 K57 and interactions of the first asparagine in the –NN– α-conotoxin motif with α3β4 I77 and I109 are key determinants of α-conotoxin activity at α3β4 nAChR. These structural insights provide a new template for the rational design of α3β4 selective inhibitors with potential as leads in the search for better treatments for nicotine addiction and lung cancer.

## Methods

### Protein expression and purification

Ls-AChBP and Ac-AChBP was over-expressed and purified as described previously^39^. Ls-AChBP was concentrated to 5 mg/mL for crystallisation trials.

### Peptide synthesis

LsIA analogues were synthesised on a rink-amide resin using Fmoc-Solid Phase Peptide Synthesis and HBTU/DIPEA activation. Final cleavage of peptide was achieved using TFA/Water/TIPS (90:5:5) solution for 3 h. Cold ether was used to precipitate peptide which was subsequently filtered. The crude peptide was lyophilised using acetonitrile/water buffer.

Peptide oxidation: After HPLC purification of crude peptides and lyophilisation the pure reduced peptides were oxidised at room temperature for a period of 18 h in 5% DMSO / NH_4_HCO_4_ (0.1 M) solution and a pH of 7.6. The oxidised peptides were analysed by LC MS indicating a uniform oxidation profile for all peptides with a single main oxidation product containing the typical α-conotoxin 1–3, 2–4 disulfide connectivity. The oxidised peptides were further purified by HPLC followed by freeze drying.

### HPLC analysis and purification

Analytical HPLC runs were performed using a Shimadzu HPLC system LC10A with a dual wavelength UV detector set at 214 nm and 254 nm. A reversed-phase C-18 column (Hypersil Gold C18, 3 μm, 100 mm x 2.1 mm) with a flow rate of 0.3 mL/min was used. Gradient elution was performed (40 °C) with the following buffer systems: **A,** 0.05% TFA in water and **B,** 0.043% TFA in 90% acetonitrile in water, from 0% **B** to 80% **B** in 20 min. Absorbance was monitored at 214 nm and 254 nm and crude purities are given by peak areas at 214 nm.Peptides were purified by preparative HPLC on a Shimadzu HPLC system on a reversed-phase C-18 column (Vydac C-18, 25 cm x 2.5 cm) at a flow rate of 15 ml/min with a 0.5% gradient of 10–60% **B**. The purity of the final products was evaluated by analytical HPLC (Hypersil C18, 130 Å, 5 μm, 250 mm × 4.6 mm, 1 ml/min flow, Gradient 10% **B** to 60% **B** in 50 min).

### Electrospray Mass Spectrometry (ESI-MS)

Electrospray mass spectra were collected inline during analytical HPLC runs using an Applied Biosystems, quadrupole spectrometer (API-150) operating in the positive ion mode with a declustering potential (DP) of 20 V, a focusing potential (FP) of 220 V and a turbospray heater temperature of 350 °C. Masses between 300 and 2200 amu were detected (Step 0.2 amu, Dwell 0.3 ms).

### Circular dichroism (CD)

CD was used to confirm the structural integrity of the chemically synthesised LsIA and the analogues. Data was recorded from 260 nm to 185 nm on a Jasco J-180 polarimeter (Jasco, Tokyo, Japan). A cell with the capacity of 400 μL and path length of 0.1 cm was used. Experiments were carried out at room temperature at a resolution of 1 nm, a scan speed of 10 min and a 4 s response time. Each spectrum was obtained from an average of three scans on 300 μg/ mL of peptide in water. The molar ellipticity was calculated and plotted against the wavelength.

### Mutagenesis

Q55K, R104A and Q73A Ls-AChBP mutants were generated using site-directed mutagenesis following the manufacturer’s instructions (QuikChange™ mutagenesis kit). Primers with the desired mutations were commercially obtained (Sigma-Aldrich). The mutated DNA was transformed into *Top10 E. coli* (Invitrogen) competent cells and isolated using a PureLink HQ Plasmid MiniPrep kit (Life Technologies). Purified DNA was used to confirm all mutations by sequencing performed at the Australian Genome Research Facility. The mutated proteins were expressed and purified as above.

### Binding assays

Competitive radioligand binding assay with ^3^H-epibatidine (specific activity 1.11–2.59 TBq/mmol) were performed as described previously^39^.

### Crystallisation

Ls-AChBP and LsIA were incubated in a molar ratio of 1:2, for one hour/ 4 °C before setting up crystallisation trials. Crystals of the Ls-AChBP and LsIA complex grew at room temperature using the hanging drop method. Orthorhombic crystals (C 2 2 2_1_) of the complex were grown in 0.8 M ammonium sulphate, 7.5% polyethylene glycol (PEG) 3350, 7.45% 2-propanol and 0.1 M ammonium acetate pH 4.3. Cell constants are: a = 115.8 Å, b = 124.5 Å, c = 154.2 Å, with 1 pentameric Ls-AChBP per asymmetric unit (asu). Clear electron density for LsIA was found in all five binding pockets.

### Structure determination and refinement

Diffraction data was collected at the MX1 beamline at the Australian Synchrotron. Data was integrated using iMOSFLM and scaled using AIMLESS^40^,^41^. The structure was solved by molecular replacement with Phaser-MR^42^ using 3ZDH as the search model. Initial refinement against experimental data was carried out using Phenix.Refine and COOT until clear electron density for LsIA was visible^43^,^44^. Most of the peptide could be autobuilt into density using Buccaneer^45^. Further refinements were carried out using Buster and COOT with NCS restraints^46^. TLS restraints, defining each subunit as a domain were applied towards the final refinements. The structure was validated using MOLPROBITY and PDB validation^47^.

### SH-SY5Y cell culture, FLIPR^TETRA^ system to measure α7 and α3β4 function

SH-SY5Y human neuroblastoma cells were cultured as described earlier^23^. Experiments were carried out over a period of several weeks and spanned on an average a minimum of 10–20 passages. Responses were not affected by passage number, with consistent control responses recorded for every experiment.

Cultured SH-SY5Y cells were seeded at 100,000 cells per well in black-walled 384 imaging plates (Corning^®^ Sigma Aldrich). Cells were seeded 48 h prior to the experiment to allow the formation of a confluent monolayer. The FLIPR^TETRA^ system was used to measure intracellular calcium increases in response to choline activating α7 and nicotine activating the α3β4 nAChRs expressed by the SH-SY5Y cells. Cells were incubated at 37 °C for 30 min, with component A of the calcium 4 assay kit (Molecular devices). The dye contains the calcium flurophore required for calcium imaging. Following incubation, the cells were transferred to the FLIPR^TETRA^ where measurements were made using a cooled CCD camera with excitation at 470–495 nm and emission at 515–575 nm. Camera gain and intensity were adjusted for each plate of cells yielding 1500–2000 arbitrary fluorescence units (AFU) as a baseline fluorescence value. LsIA and analogues were added 10 mins before applying choline (for α7) or nicotine (for α3β4) (30 μM). Additionally, N-(5-Chloro-2,4-dimethoxyphenyl)-N′-(5-methyl-3-isoxazolyl)-urea (PNU-120596) is also used (10 μM) to measure activity at the α7 subtype on the FLIPR platform. The channel kinetics are too fast to measure otherwise.

### Molecular modelling

Homology models were generated using the project mode of the SWISSMODEL online server. Briefly, the FASTA sequences for the ligand binding domain of the nAChRs were loaded into the DEEPVIEW program. The crystal structure of the Ls-AChBP and LsIA complex was then loaded and its sequence automatically aligned with that of the nAChRs. Manual adjustments were required to improve quality of alignment. The resulting model was energy minimised using the GROMOS force field in the program DEEPVIEW and models visualised in PyMol.

### Structure analysis

Receptor-ligand interactions were analysed using PDBsum, QtPISA v1.18 and manual inspection in PyMol. Bond distances were measured in all five binding pockets and expressed as a mean ± S.E.M.

### Data analysis

Radioligand binding data were evaluated by a nonlinear, least squares one-site competition fitting procedure using GraphPad Prism 6.0 (GraphPad Software Inc., San Diego, CA, USA). FLIPR data was normalised to the maximum choline or nicotine (10 μM or 100 μM where indicated) response in the SH-SY5Y cells to yield the %F_max_. A four-parameter Hill equation was fitted to the data using GraphPad Prism 6.0. The Hill-slope was fixed to -1 where the 95% confidence intervals spanned this value. Experiments were performed in triplicates in three independent experiments. IC_50_ values are reported as mean ± S.E.M.

## Additional Information

**Accession codes:** Coordinates and structure factors for the LsIA-LsAChBP complex have been deposited in the RCSB PDB with ID 5T90.

**How to cite this article:** Abraham, N. *et al*. Structural mechanisms for a-conotoxin activity at the human a3b4 nicotinic acetylcholine receptor. *Sci. Rep.* **7**, 45466; doi: 10.1038/srep45466 (2017).

**Publisher's note:** Springer Nature remains neutral with regard to jurisdictional claims in published maps and institutional affiliations.

## Supplementary Material

Supplementary Information

## Figures and Tables

**Table 1:** α-Conotoxins that inhibit α3β4 nAChR.

α-conotoxin	Sequence	nAChR selectivity (nM)	Ref.
LsIA	SGCCSNPACRVNNPNIC^*^	α7(10.1) ≥ α3β2 (10.3), **α3β4** (inactive)	23
RegIIA	-GCCSHPACNVNNPHIC^*^	α3β2(33) > **α3β4(97)** > α7(103) > α4β2(>1,000) > α9α10(1,000)	24
Mr 1.1	-GCCSHPACSVNNPDIC^*^	α3β2 (61%) > α7 (~55%) > **α3β4 (40%)**^#^	32
PeIA	-GCCSHPACAGNNQHIC^*^	α9α10 (6.9) > α3β2 (23) > **α3β4 (**480) > α7 (1,800) > α4β2 (11,600)	27
GIC	-GCCSHPACAGNNQHIC^*^	α3β2 (1.1) > α4β2 (309) > **α3β4 (**775)	28
BuIA	-GCCSTPPCAVLY—C^*^	α6/α3β2 (0.26) > α6/α3β4 (1.54) > α3β2 (5.72) > > **α3β4 (**27.7) > α4β4 (69.9) > α2β4 (121) > α7 (272) > α2β2 (800) > α4β2 (10,400).	26
AuIB	-GCCSYPPCFATNPD-C^*^	**α3β4 (**750) > α7, α4β4, α3β2, α4β2, α2β2, α2β4 (>1,000)	29
TxID	-GCCSHPVCSAMSP-IC^*^	**α3β4 (**12.5) > α6α3β4 (94.1) > α2β4 (4,550) > α4β4, α4β2, α6/α3β32β3, α3β2, α2β2,α9α10, α7 (>10,000)	30
TP-2212–59	-GCCSHPBCFBZY—C^*^	**α3β4 (**2.3) > α7 (>1,000), α3β2 (1,000)	31

B is 2-aminobutyric acid (Abu), Z is norvaline (Nva), and (*) indicates C-terminal amidation. ^#^Authors do not report an IC_50_ instead report % inhibition of Ach-evoked currents.

**Table 2:** IC_50_ values for displacement of ^3^H-epibatidine binding and inhibition of nAChR current in SH-SY5Y cells by LsIA and analogues.

Ligand	^3^H-epibatidine (IC_50_ ± S.E.M)			SH-SY5Y (IC_50_ ± S.E.M)	
	Ls-AChBP (μM)	Ac-AChBP (nM)	Q55K-LsAChBP (μM)	α7 (μM)	α3β4 (μM)
LsIA	0.21 ± 0.04	5.44 ± 0.35	17.4 ± 10.7	0.07 ± 0.007	>10
N6H-LsIA	ND	ND	ND	0.04 ± 0.002	>10
R10M-LsIA	0.50 ± 0.16	1.88 ± 0.65	7.1 ± 2.5	2.42 ± 1.03	0.44 ± 0.13
R10D-LsIA	>10	8,372 ± 3	>10	>10	>10
R10F-LsIA	0.11 ± 0.02	2.20 ± 0.05	0.8 ± 0.26	0.41 ± 0.41	0.44 ± 0.13
N12Q-LsIA	2.44 ± 0.75	55.2 ± 0.9	ND	0.98 ± 0.61	>10
N12D-LsIA	>10	5,546 ± 212	ND	>10	>10
N12L-LsIA	>10	61 ± 7.0	ND	25.7 ± 12.3	0.63 ± 0.16
[R10F][N12L]-LsIA	ND	ND	ND	>100	0.41 ± 0.02

ND = not determined.
