# Supplementary material for: Structural mechanisms for α-conotoxin activity at the human α3β4 nicotinic acetylcholine receptor
Source: Sci Rep. 2017 Mar 31;7:45466. doi: 10.1038/srep45466 (PMC5374441; doi:10.1038/srep45466)
Supplement: Supplementary Information [file srep45466-s1.pdf]

Structural mechanisms for α-conotoxin activity at the human α3β4 nicotinic acetylcholine receptor

Nikita Abraham, Michael Healy, Lotten Ragnarsson, Andreas Brust,

Paul Alewood, Richard Lewis*

Supplementary information

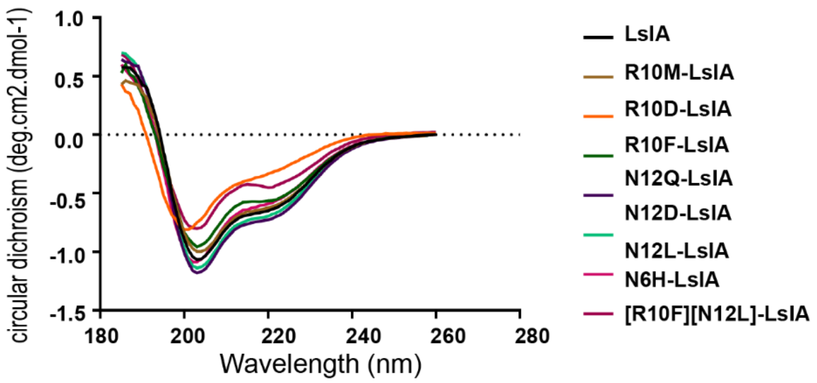

Supplementary figure S1: CD spectra for synthetic LsIA analogues. LsIA analogues with modifications to position 6, 10 and 12 were chemically synthesised. R10M, R10D and R10F were synthesised as analogues of position 10. N12Q, N12D and N12L as analogues for position 12. CD spectra for LsIA analogues confirmed that all the analogues preserved the α-helical fold, similar to the native peptide with the exception of R10D-LsIA where the peak at 222 nm was shallower and the peak at 204 nm shifted to 200 nm as compared to LsIA. Suggesting that R10D-LsIA conformation might differ from the conformation of native LsIA, possibly due to global structural changes in the peptide induced by this modification.

```
Ls-AChBP      ----------LDRADILYNIRQTSRPDVIPTQRDRPVAVSVSLKFINILEVNEITNEVDV  50
hα3           --------AEHRLFERLF--EDYNEIIRPVANVSDPVIIHFEVSMSQLVKVDEVNQIMET  50
rα3           --------AEHRLFQYLF--EDYNEIIRPVANVSHPVIIQFEVSMSQLVKVDEVNQIMET  50

Ls-AChBP      VFWQQTTWSDRTLAWNSSHSP--DQVSVPISSLWVPDLAAYNAIS-KPEVLTPQLARVVS  107
hα3           NLWLKQIWNDYKLKWNPSDYGGAEFMRVPAQKIWKPDIVLYNNAVGDFQVDDKTKALLKY  110
rα3           NLWLKQIWNDYKLKWKPSDYQGVEFMRVPAEKIWKPDIVLYNNADGDFQVDDKTKALLKY  110

Ls-AChBP      DGEVLYMPSIRQRFSCDVSGVD-TESGATCRIKIGSWTHHSREISVDPTTENSDDSEYFS  166
hα3           TGEVTWIPPAIFKSSCKIDVTYFPFDYQNCTMKFGSWSYDKAKIDLVLIGSSMNLKDYW-  169
rα3           TGEVTWIPPAIFKSSCKIDVTYFPFDYQNCTMKFGSWSYDKAKIDLVLIGSSMNLKDYW-  169

Ls-AChBP      QYSRFEILDVTQKKNSVTYSCCPEAYEDVEVSLNFRKKGRSEIL- 210
hα3           ESGEWAIIKAPGYKHDIKYNCCEEIYPDITYSLYIRRLPLFYTIN 214
rα3           ESGEWAIIKAPGYKHEIKYNCCEEIYQDITYSLYIRRLPLFYTI- 213
```

Supplementary figure S2: Sequence alignment for the human and rat α3 subunit ligand binding domain. The human (Uniprot: P32297) and rat (Uniprot: P04757) α3 are aligned with the Ls-AChBP. The α3 subunit from the two species share 95% sequence identity with an E value of $1x10^{-150}$. The residues varying between the two species are highlighted in red. The residues participating in receptor-ligand interactions are bold with grey back ground.

```
Ls-AChBP      ----LDRADILYNIRQTSRPDVIPTQRDRPVAVSVSLKFINILEVNEITNEVDVVFWQQT  56
hβ2           TDTEERLVEHLLDPSRYNKLIRPATNGSELVTVQLMVSLAQLISVHEREQIMTTNVWLTQ  60
rβ2           --TEERLVEHLLDPSRYNKLIRPATNGSELVTVQLMVSLAQLISVHEREQIMTTNVWLTQ  58

Ls-AChBP      TWSDRTLAWNSSHSP--DQVSVPISSLWVPDLAAYNAIS-KPEVLTPQLARVVSDGEVLY  113
hβ2           EWEDYRLTWKPEEFDNMKKVRLPSKHIWLPDVVLYNNADGMYEVSFYSNAVVSYDGSIFW  120
rβ2           EWEDYRLTWKPEEFDNMKKVRLPSKHIWLPDVVLYNNADGMYEVSFYSNAVVSYDGSIFW  118

Ls-AChBP      MPSIRQRFSCDVSGVD-TESGATCRIKIGSWTHHSREISVDPTTENSDDSEYFSQYSRFE  172
hβ2           LPPAIYKSACKIEVKHFPFDQQNCTMKFRSWTYDRTEIDLVLKSEVASLDDFTP-SGEWD  179
rβ2           LPPAIYKSACKIEVKHFPFDQQNCTMKFRSWTYDRTEIDLVLKSEVASLDDFTP-SGEWD  177

Ls-AChBP      ILDVTQKKNSVTYSCCPE-AYEDVEVSLNFRKKGRSEIL--  210
hβ2           IVALPGRRNENPD----DSTYVDITYDFIIRRKPLFYTI-  214
rβ2           IIALPGRRNENPD----DSTYVDITYDFIIRRKP------  207
```

Supplementary figure S3: Sequence alignment for the human and rat β2 subunit ligand binding domain. The human (Uniprot: P11787) and rat (Uniprot: P12390) β2 are aligned with the Ls-AChBP. The β2 subunit from the two species share 99% sequence identity with an E value of $2x10^{-158}$. The residues participating in receptor-ligand interactions are highlighted with grey back ground.

```
Ls-AChBP      ---LDRADILYNIRQTSRPDVIPTQRDRPVAVSVSLKFINILEVNEITNEVDVVFWQQTT 57
hβ4           -AEEKLMDDLLNKTRYNNLIRPATSSSQLISIKLQLSLAQLISVNEREQIMTTNVWLKQE 59
rβ4           -AEEKLMDDLLNKTRYNNLIRPATSSSQLISIRLELSLSQLISVNEREQIMTTSIWLKQE 59

Ls-AChBP      WSDRTLAWNSSHSP--DQVSVPISSLWVPDLAAYNAIS-KPEVLTPQLARVVSDGEVLYM 114
hβ4           WTDYRLTWNSSRYEGVNILRIPAKRIWLPDIVLYNNADGTYEVSVYTNLIVRSNGSVLWL 119
rβ4           WTDYRLAWNSSCYEGVNILRIPAKRVWLPDIVLYNNADGTYEVSVYTNVIVRSNGSIQWL 119

Ls-AChBP      PSIRQRFSCDVSGVD-TESGATCRIKIGSWTHHSREISVDPTTENSDDSEYFSQYSRFEI 173
hβ4           PPAIYKSACKIEVKYFPFDQQNCTLKFRSWTYDHTEIDMVLMTPTASMDDFTP-SGEWDI 178
rβ4           PPAIYKSACKIEVKHFPFDQQNCTLKFRSWTYDHTEIDMVLKSPTAIMDDFTP-SGEWDI 178

Ls-AChBP      LDVTQKKNSVTYSCCPE-AYEDVEVSLNFRKKGRSEIL--      210
hβ4           VALPGRRTVNPQ----DPSYVDVTYDFIIKRKPLFYTI        212
rβ4           VALPGRRTVNPQ----DPSYVDVTYDFIIKRKPLFYTI        212
```

Supplementary figure S4: Sequence alignment for the human and rat β4 subunit ligand binding domain. The human (Uniprot: P30926) and rat (Uniprot: P12392) β4 are aligned with the Ls-AChBP. The β4 subunit from the two species share 93% sequence identity with an E value of $4x10^{-154}$. The residues varying between the two species are highlighted in red. The residues participating in receptor-ligand interactions are highlighted in grey back ground.

```
Ls-AChBP     --LDR---ADILYNIRQTSRPDVIPTQRDRPVAVSVSLKFINILEVNEITNEVDVVFWQQ 55
hα7          GEFQRKLYKELVKNYNPLERP---VANDSQPLTVYFSLSLLQIMDVDEKNQVLTTNIWLQ 57
rα7          GEFQRRLYKELVKNYNPLERP---VANDSQPLTVYFSLSLLQIMDVDEKNQVLTTNIWLQ 57

Ls-AChBP     TTWSDRTLAWNSSHSP--DQVSVPISSLWVPDLAAYNAIS-KPEVLTPQLARVVSDGEVL 112
hα7          MSWTDHYLQWNVSEYPGVKTVRFPDGQIWKPDILLYNSADERFDATFHTNVLVNSSGHCQ 117
rα7          MSWTDHYLQWNMSEYPGVKNVRFPDGQIWKPDILLYNSADERFDATFHTNVLVNASGHCQ 117

Ls-AChBP     YMPSIRQRFSCDVSGV-DTESGATCRIKIGSWTHHSREISVDPTTENSDDSEYFSQYSRF 171
hα7          YLPPGIFKSSCYIDVRWFPFDVQHCKLKFGSWSYGGWSLDLQM--QEADISGYIP-NGEW 174
rα7          YLPPGIFKSSCYIDVRWFPFDVQQCKLKFGSWSYGGWSLDLQM--QEADISSYIP-NGEW 174

Ls-AChBP     EILDVTQKKNSVTYSCCPEAYEDVEVSLNFRKKGRSEIL     210
hα7          DLVGIPGKRSERFYECCKEPYPDVTFTVTMRRRT-----     208
rα7          DLMGIPGKRNEKFYECCKEPYPDVTYTVTMRRRT-----     208
```

Supplementary figure S5: Sequence alignment for the human and rat α7 subunit ligand binding domain. The human (Uniprot: P30926) and rat (Uniprot: P12392) α7 are aligned with the Ls-AChBP. The α7 subunit from the two species share 95% sequence identity with an E value of $5x10^{-156}$. The residues varying between the two species are highlighted in red. The residues participating in receptor-ligand interactions are highlighted in bold with grey back ground (residues of the principle face) and in *italics* with grey background (residues of the complimentary face).

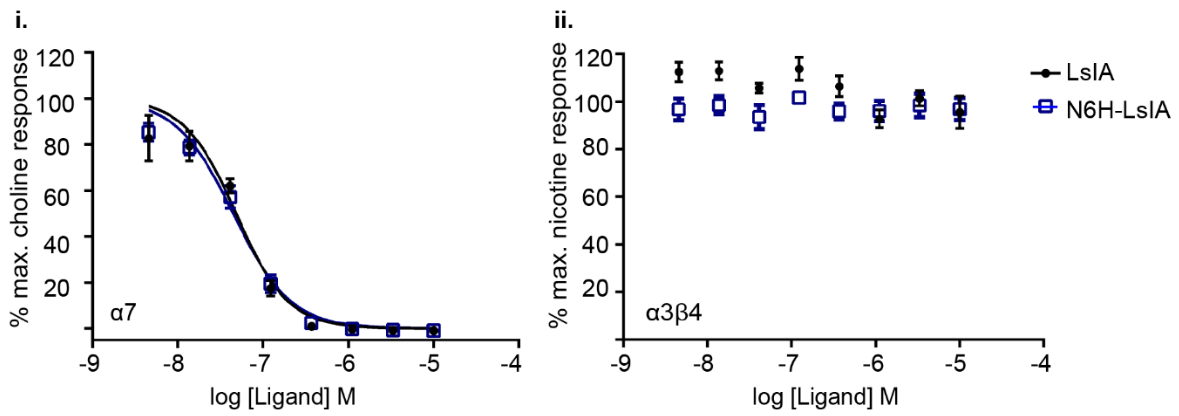

Supplementary figure S6: Characterisation of LsIA-N6H analogue. A histidine residue at position 6 is a common feature for α-conotoxins that antagonise the α3β4 nAChR. LsIA N6 was substituted with histidine. The N6H analogue had a functional profile similar to LsIA at α7 and α3β4 subtypes. This suggests that the histidine residue is not critical for the activity and selectivity of antagonists at the α3β4 nAChR.

```
Ls-AChBP      ------LDRADILYNIRQTSRPDVIPTQRDRPVAVSVSLKFINILEVNEITNEVDVVFWQ 54
Ac-AChBP      HSQANLMRLKSDLFN----RSPMYPGPTKDDPLTVTLGFTLQDIVKADSSTNEVDLVYYE 56
hα7           ----FQRKLYKELV--KNYNPLERPVANDSQPLTVYFSLSLLQIMDVDEKNQVLTTNIWL 54
hα3           ----AEHRLFERLF--EDYNEIIRPVANVSDPVIIHFEVSMSQLVKVDEVNQIMETNLWL 54
hβ2           --TDTEERLVEHLLDPSRYNKLIRPATNGSELVTVQLMVSLAQLISVHEREQIMTTNVWL 58
hβ4           ----AEEKLMDDLLNKTRYNNLIRPATSSSQLISIKLQLSLAQLISVNEREQIMTTNVWL 56

              *                   *                               *
Ls-AChBP      QTTWSDRTLAWNSSHSP--DQVSVPISSLWVPDLAAYNAIS-KPEVLTPQLARVVSDGEV 111
Ac-AChBP      QQRWKLNSLMWDPNEYGNITDFRTSAADIWTPDITAYSSTR-PVQVLSPQIAVVTHDGSV 115
hα7           QMSWTDHYLQWNVSEYPGVKTVRFPDGQIWKPDILLYNSADERFDATFHTNVLVNSSGHC 114
hα3           KQIWNDYKLKWNPSDYGGAEFMRVPAQKIWKPDIVLYNNAVGDFQVDDKTKALLKYTGEV 114
hβ2           TQEWEDYRLTWKPEEFDNMKKVRLPSKHIWLPDVVLYNNADGMYEVSFYSNAVVSYDGSI 118
hβ4           KQEWTDYRLTWNSSRYEGVNILRIPAKRIWLPDIVLYNNADGTYEVSVYTNLIVRSNGSV 116

                                               *                   *
Ls-AChBP      LYMPSIRQRFSCDVSGVD-TESGATCRIKIGSWTHHSREISVDPTTENSDDSEYFSQYSR 170
Ac-AChBP      MFIPAQRLSFMCDPTGVD-SEEGATCAVKFGSWVYSGFEIDLKTDTDQVDLSSYYA-SSK 173
hα7           QYLPPGIFKSSCYIDVRWFPFDVQHCKLKFGSWSYGGWSLDLQMQE--ADISGYIP-NGE 171
hα3           TWIPPAIFKSSCKIDVTYFPFDYQNCTMKFGSWSYDKAKIDLVLIGSSMNLKDYWE-SGE 173
hβ2           FWLPPAIYKSACKIEVKHFPFDQQNCTMKFRSWTYDRTEIDLVLKSEVASLDDFTP-SGE 177
hβ4           LWLPPAIYKSACKIEVKYFPFDQQNCTLKFRSWTYDHTEIDMVLMTPTASMDDFTP-SGE 175

                            *    *  *
Ls-AChBP      FEILDVTQKKNSVTYSCCPE-AYEDVEVSLNFRKKGRSEIL------     210
Ac-AChBP      YEILSATQTRQVQHYSCCPE-PYIDVNLVVKFRERRAGNGFFRNLFD     219
hα7           WDLVGIPGKRSERFYECCKE-PYPDVTFTVTMRRRT-----------     206
hα3           WAIIKAPGYKHDIKYNCCEE-IYPDITYSLYIRRLPLFYTIN-----     214
hβ2           WDIVALPGRRNENPD----DSTYVDITYDFIIRRKPLFYTI------     214
hβ4           WDIVALPGRRTVNPQ----DPSYVDVTYDFIIKRKPLFYTI------     212
```

Supplementary figure S7: Multiple sequence alignment of the Ac- , Ls-AChBPs, human α7, α3,β2 and β4 subunits. The alignment highlights residues involved in receptor-ligand interactions. Principle side contacts are in bold with grey background. Complimentary side contacts are in italics with grey background. Residues involved in hydrogen bond are indicated with (*), Residues not in direct contact, but are a part of the interacting surface are boxed.

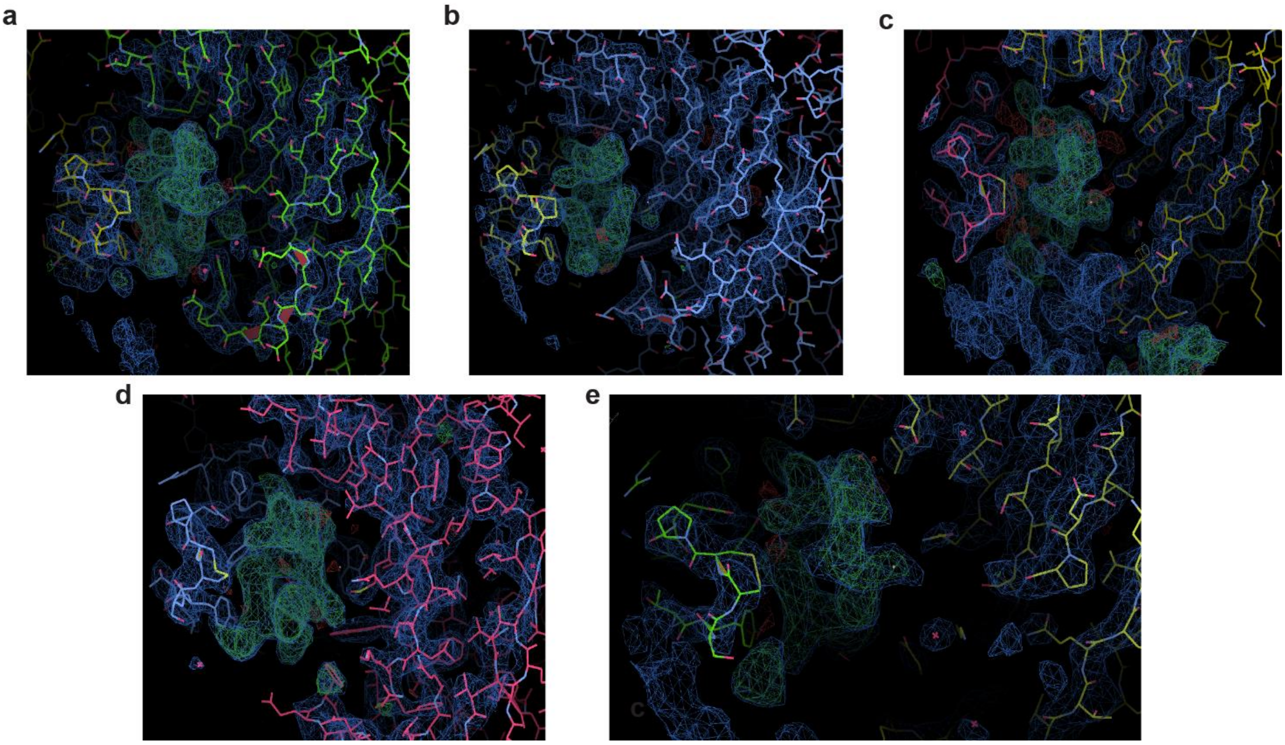

Supplementary figure S8: Electron density maps of the LsIA/Ls-AChBP co-crystal structure. Shown are the Fo-Fc (3.0 σ) and 2Fo-Fc (1.0 σ) maps for the co-crystal structure before peptide building. The ligand binding pocket shows clear electron density for LsIA. The five interfaces are shown as (a) Subunits A-E interface (b) Subunits B-D (c) Subunits C-A (d) Subunits D-C (e) Subunits E-B.

```
Ls-AChBP      ---------LDRADILYNIRQTSRPDVIPTQRDRPVAVSVSLKFINILEVNEITNEV    48
Ac-AChBP      ---HSQANLMRLKSDLFN----RSPMYPGPTKDDPLTVTLGFTLQDIVKADSSTNEV    50
hα7           -------FQRKLYKELV--KNYNPLERPVANDSQPLTVYFSLSLLQIMDVDEKNQVL    48
hβ2           -----TDTEERLVEHLLDPSRYNKLIRPATNGSELVTVQLMVSLAQLISVHEREQIM    52
hβ4           -------AEEKLMDDLLNKTRYNNLIRPATSSSQLISIKLQLSLAQLISVNEREQIM    50
hα3           -------AEHRLFERLF--EDYNEIIRPVANVSDPVIIHFEVSMSQLVKVDEVNQIM    48
hα4           HVETRAHAEERLLKKLF--SGYNKWSRPVANISDVVLVRFGLSIAQLIDVDEKNQMM    55

Ls-AChBP      DVVFWQQTTWSDRTLAWNSSHSP--DQVSVPISSLWVPDLAAYNAIS-KPEVLTPQLARV 105
Ac-AChBP      DLVYYEQQRWKLNSLMWDPNEYGNITDFRTSAADIWTPDITAYSSTR-PVQVLSPQIAVV 109
hα7           TTNIWLQMSWTDHYLQWNVSEYPGVKTVRFPDGQIWKPDILLYNSADERFDATFHTNVLV 108
hβ2           TTNVWLTQEWEDYRLTWKPEEFDNMKKVRLPSKHIWLPDVVLYNNADGMYEVSFYSNAVV 112
hβ4           TTNVWLKQEWTDYRLTWNSSRYEGVNILRIPAKRIWLPDIVLYNNADGTYEVSVYTNLIV 110
hα3           ETNLWLKQIWNDYKLKWNPSDYGGAEFMRVPAQKIWKPDIVLYNNAVGDFQVDDKTKALL 108
hα4           TTNVWVKQEWHDYKLRWDPADYENVTSIRIPSELIWRPDIVLYNNADGDFAVTHLTKAHL 115

Ls-AChBP      VSDGEVLYMPSIRQRFSCDVSGVD-TESGATCRIKIGSWTHHSREISVDPTTENSDDSEY 164
Ac-AChBP      THDGSVMFIPAQRLSFMCDPTGVD-SEEGATCAVKFGSWVYSGFEIDLKTDTDQVDLSSY 167
hα7           NSSGHCQYLPPGIFKSSCYIDVRWFPFDVQHCKLKFGSWSYGGWSLDLQMQE--ADISGY 165
hβ2           SYDGSIFWLPPAIYKSACKIEVKHFPFDQQNCTMKFRSWTYDRTEIDLVLKSEVASLDDF 171
hβ4           RSNGSVLWLPPAIYKSACKIEVKYFPFDQQNCTLKFRSWTYDHTEIDMVLMTPTASMDDF 169
hα3           KYTGEVTWIPPAIFKSSCKIDVTYFPFDYQNCTMKFGSWSYDKAKIDLVLIGSSMNLKDY 167
hα4           FHDGRVQWTPPAIYKSSCSIDVTFFPFDQQNCTMKFGSWTYDKAKIDLVNMHSRVDQLDF 174

Ls-AChBP      FSQYSRFEILDVTQKKNSVTYSCCPEAYEDVEVSLNFRKKGRSEIL-      210
Ac-AChBP      YA-SSKYEILSATQTRQVQHYSCCPE-PYIDVNLVVKFRERRAGNGF     213
hα7           IP-NGEWDLVGIPGKRSERFYECCKEPYPDVTFTVTMRRRT------      206
hβ2           TP-SGEWDIVALPGRRNENPD---DSTYVDITYDFIIRRKPLFYTI-      214
hβ4           TP-SGEWDIVALPGRRTVNPQ---DPSYVDVTYDFIIKRKPLFYTI-      212
hα3           WE-SGEWAIIKAPGYKHDIKYNCCEEIYPDITYSLYIRRLPLFYTIN      214
hα4           WE-SGEWVIVDAVGTYNTRKYECCAEIYPDITYAFVIRRLPLFYTIN      221
```

Supplementary figure S9: Residues modulating α-conotoxin activity at nAChRs. Residues influencing α-conotoxin activity at the α7[1] (green), α3β2[1,2] (yellow), α4β2[3,4] (bold)and α3β4[this study] (blue) are shown. Distinct sets of residues influence α-conotoxin activity at the different subtypes. A comparison of residues equivalent to the α3β4 pharmacophore in other nAChRs (boxed) reveal some sequence variations. Therefore, in addition to being primary determinants of α3β4 activity, the β4 triad identified is likely to influence the selectivity of α-conotoxins for different nAChR subtypes.

Supplementary Table S1: Data collection and refinement statistics.

	**Ls-AChBP - LsIA**
**Data collection**	
Space group	C 2 2 2_1
Cell dimensions, Å	a=115.8Å, b=124.5Å, c=154.2Å
Cell dimensions, °	α=90^0, β=90^0, γ=90^0
Resolution, Å	51.40-2.80 (2.95 - 2.80)
Rsym	0.137 (0.934)
I/Iσ	14.6 (2.6)
Completeness (%)	96.9 (90.7)
Multiplicity	11.4 (11.2)
Total no. of reflections	306906 (40559)
Unique reflections	26929 (3618)
**Refinement**	
Resolution, Å	48.44-2.8
Rwork/Rfree	0.2176 / 0.2451
rmsd bond distance, Å	0.008
rmsd bond angle, Å	1.06
Average B-factor	70.0

Supplementary table S2: Receptor-ligand interactions seen in LsIA/Ls-AChBP co-crystal structure.

**LsIA**	**Ls AChBP**	
	Principal subunit (+)	Complimentary subunit (-)
**Ser1**	Ser186#* Cys187*	
**Gly2**	**Tyr185#**	
**Cys3**	Cys187 Cys188	-
**Cys4**		**Tyr164*#**
**Ser5**		Glu163#*
**Asn6**	**Tyr185#**	
**Pro7**	Tyr89* Trp143	Trp53* Met114*
**Ala8**	**Tyr192#** Ser142 Trp143	
**Cys9**	Cys187 Cys188 Tyr192#	
**Arg10**		**Tyr164*** Ser32* **Gln55*** Lys34*
**Val11**		Leu112
**Asn12**	**Thr144* (backbone)**	**Gln73* Arg104***
**Asn13**	**Glu190 Tyr192***	
**Pro14**		Leu112*
**Asn15**		
**Ile16**	Cys187	
**Cys17**		

(#) interactions seen in ≤ 3 pockets.
(**Bold**) Hydrogen bonds (*) Interactions unique to LsIA/Ls-AChBP complex.

Supplementary references

1 Hopping, G. *et al.* Hydrophobic residues at position 10 of α-conotoxin PnIA influence subtype selectivity between α7 and α3β2 neuronal nicotinic acetylcholine receptors. *Biochemical pharmacology* **91**, 534-542 (2014).

2 Lin, B. *et al.* From crystal structure of α-conotoxin GIC in complex with Ac-AChBP to molecular determinants of its high selectivity for α3β2 nAChR. *Scientific reports* **6** (2016).

3 Beissner, M. *et al.* Efficient binding of 4/7 α-conotoxins to nicotinic α4β2 receptors is prevented by Arg185 and Pro195 in the α4 subunit. *Molecular pharmacology* **82**, 711-718 (2012).

4 Dutertre, S., Nicke, A. & Lewis, R. J. β2 subunit contribution to 4/7 α-conotoxin binding to the nicotinic acetylcholine receptor. *Journal of Biological Chemistry* **280**, 30460-30468 (2005)
